# Clonal Propagation and Assessment of Biomass Production and Saponin Content of Elite Accessions of Wild *Paris polyphylla* var. *yunnanensis*

**DOI:** 10.3390/plants12162983

**Published:** 2023-08-18

**Authors:** Mulan Wang, Weiqi Li, Qi Qiang, Junchao Ma, Jiaqi Chen, Xudong Zhang, Yanxia Jia, Tie Zhang, Liang Lin

**Affiliations:** 1 Germplasm Bank of Wild Species, Kunming Institute of Botany, Chinese Academy of Sciences, Kunming 650201, China; wangmulan@mail.kib.ac.cn (M.W.); qiangqi@hainanu.edu.cn (Q.Q.); majunchao@mail.kib.ac.cn (J.M.); chenjiaqi@mail.kib.ac.cn (J.C.); zhangxudong@mail.kib.ac.cn (X.Z.); jiayanxia@mail.kib.ac.cn (Y.J.); 2 Science and Technology Department, Wenshan University, Wenshan 663000, China

**Keywords:** medicinal plant, *Paris polyphylla* var. *yunnanesis*, somatic embryogenesis, premium cultivars

## Abstract

*Paris polyphylla* var. *yunnanensis* is an endangered medicinal plant endemic to China with great economic importance for the pharmaceutical industry. Two significant barriers to its commercial development are the long duration of its seed germination and the frequency of interspecific hybridization. We developed a method for clonal propagation of *Paris polyphylla* var. *yunnanensis* and successfully applied it to selected elite wild plants, which could become cultivar candidates based on their biomass production and saponin content. In comparison to the traditional method, somatic embryogenesis produced an average of 63 somatic embryos per gram of callus in just six weeks, saving 12 to 15 months in plantlet production. The produced in vitro plantlets were strong and healthy and 94% survived transplanting to soil. Using this method, four candidate cultivars with diverse morphologies and geographic origins were clonally reproduced from selected elite wild accessions. In comparison to those obtained with the traditional *P. polyphylla* propagation technique, they accumulated higher biomass and polyphyllin levels in rhizomes plus adventitious roots during a five-year period. In conclusion, somatic embryogenesis-based methods offer an alternate approach for the rapid and scaled-up production of *P. polyphylla*, as well as opening up species conservation options.

## 1. Introduction

*Paris polyphylla Smith* var. *yunnanensis* (Franch.) Hand.-Mazz is a perennial herb belonging to the Melanthiaceae family. Its rhizome (Rhizoma Paridis) or active ingredient polyphyllin are used as raw materials for ~70 commercial drugs and health products. Well-known Chinese medications that are made from Rhizoma Paridis extract include Yunnan Baiyao and Gongxuening capsule, both of which are used for gynecological hemostasis. Polyphyllin has been shown to have analgesic, anti-inflammatory, hemostasis, and anticancer activities [1]. Polyphyllin I (PPI), II (PPII), and VII (PPVII) are documented in the latest (2020) edition of the Chinese Pharmacopoeia [2]. In China, the pharmaceutical sector utilizes over 3,000,000 kg of raw *P. polyphylla* annually, and the market price of Rhizoma Paridis has increased 100 times in the past 30 years, reaching CNY 650 (approximately USD 100)/kg in 2019 [3]. However, it has been found that *P. polyphylla* has two physiological traits that are incompatible with the innovation and development requirements of the pharmaceutical industry for which this plant is cultivated.

One of the problem physiological traits in *P. polyphylla* is the extremely long period of seed dormancy-release and germination. Seeds require more than 18 months of treatment to break dormancy. As this can extend over two winter seasons, the seeds are said to have “double dormancy”, which needs to be overcome for the active growth of the root and shoot. Overharvesting for the huge industrial demand in recent decades has resulted in smaller population sizes. When reduced reproductive output is combined with slow regeneration due to double dormancy, reproductive efficiency is lowered. This affects not only the economic efficiency of the planting industry but also the species’ survival, and its genetic resource, in the wild. *P. polyphylla* has been listed as a rare and endangered plant species in Yunnan province [4].

The second trait that impacts the species’ economic development is the high capacity for interspecific hybridization in the genus *Paris*. In China, there are 22 species or subspecies in the genus, distributed from the northeast to southwest [5]. However, only two of them have been listed in the Chinese pharmacopoeia for pharmaceutical use. Unintended hybridization between *P. polyphylla* and other species often results in variations in the wild-type species that are difficult to distinguish from morphological characteristics. This can damage the uniformity of *P. polyphylla* as medicinal raw material and frustrate seed-based breeding work. This problem can be technically solved by rhizome cutting, a traditional clonal propagation method for this and other species. However, this propagation technique either vastly reduces net rhizome yields or results in a low multiplication rate because of limited axillary meristems [6,7]. Additionally, it has been observed that the cutting of rhizomes makes them susceptible to attack by insects and diseases. Rhizome cuts can lead to extensive rotting and substantial yield losses [8,9]. To address these limitations of conventional propagation of *P. polyphylla*, we developed a novel strategy for in vitro-based multiplication.

Somatic embryogenesis (SE) is widely considered to be a highly efficient micropropagation system and is now widely used within plant-based industries [10,11,12,13]. It can generate genetically uniform plant lines that are free of undesirable characteristics in a short period of time. Furthermore, it can be used to generate new cultivars from selected plants. For example, SE has been successfully used for large-scale propagation of elite oil palm lines with a proliferation rate of 10,000 embryos per liter [12,14]. In the case of *Echinacea purpurea* (L.) Moench’s, antioxidant content was increased using SE propagation, which eliminated the need for labor-intensive and time-consuming breeding efforts [15]. However, to the best of our knowledge, there is no innovation platform yet for the generation and use of SE for *P. polyphylla*.

In this study, we report an SE-based production method for *P. polyphylla* plantlets. It can produce plantlets at large scale and significantly reduce the period from cultivation through to planting using traditional (macro) propagation methods. Monitoring of the plantlet emergence during nursery growth, detailing of the growth conditions at different stages, the development of plants, and the determination of the levels of active ingredients all showed that this system has great potential for the planting industry. Four candidate species for cultivar development were also propagated and screened successfully.

## 2. Results

### 2.1. Production of Somatic Embryos and Their Conversion to Plantlets

**Figure 1:**
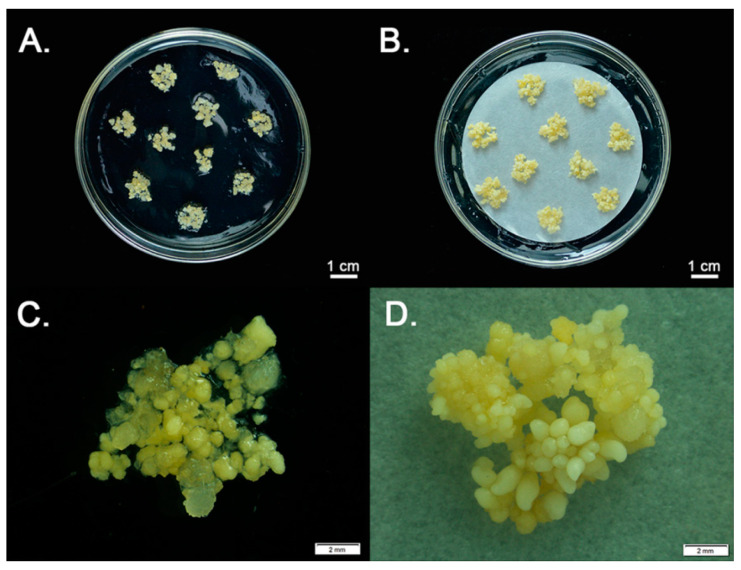
Somatic embryo innovation platform for *Paris polyphylla***.** Somatic embryos grown on medium (**A**,**C**) compared with stronger growth on filter paper + medium (**B**,**D**).

The successfully established *P. polyphylla* somatic embryos are shown in Figure 1. When differentiated from transparent, yellow embryogenic calli, the somatic embryos had a granular structure and were yellow in color. The somatic embryos proliferated more on “filter paper + medium” than on medium alone. With 1 g of callus, we were able to induce an average of 63 somatic embryos, whereas in previous research, we could only create 9 normal embryos from the same callus weight (Figure 1 and Table S1). These findings indicate that, in terms of economies of scale, a commercial production method for *P. polyphylla* plants could be developed using an SE-based approach.

**Figure 2:**
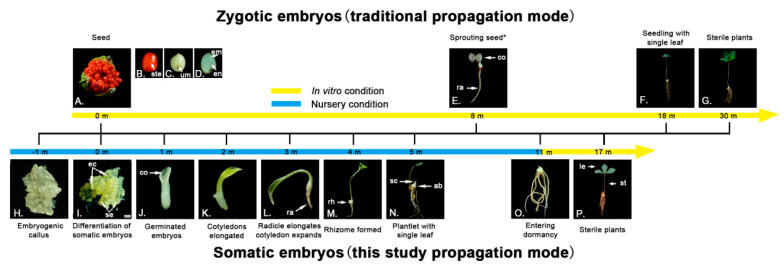
Different stages during *P. polyphylla* germination and plantlet regeneration from zygotic embryos (top panel) and somatic embryos (bottom panel). Plantlets and seedlings refer to the plants derived from tissue culture and seeds, respectively. (**A**) Dehisced capsule and seeds of *P. polyphylla*. (**B**) Seeds wholly covered with red, fresh, and juicy sarcotesta. (**C**) Umbilici borne at the seed base. (**D**) The undifferentiated embryo within the endosperm occupies a small area of the seed. (**E**) The embryo grows gradually and the radicle first protrudes from the micropyle. The asterisk signifies that some of the dwarf accessions would germinate within three months of planting, but most would not germinate until eight months later. (**F**) The cotyledon breaks away from the seed coat and spreads out to form the first heart-shaped leaf. (**G**) Single-leaf seedlings become sterile plants, which bear a whorl of leaves (three to five) at the stem apex. (**H**) Translucent yellow embryogenic callus. (**I**) Initiation of a large number of somatic embryos from the embryogenic callus. (**J**) The apex of the globular embryos is further differentiated. (**K**) Cotyledonary embryos are formed. (**L**) The cotyledonary embryo and radicle become elongated to generate a complete plantlet with well-developed roots and shoots. (**M**) The base of the hypocotyl connecting the root of the plant to the stem segment is enlarged and oval. (**N**) Axillary buds are formed in the rhizome, and the scales (leaf sheaths) on the surface of the rhizome are raised in the shape of a spire. (**O**) The plant enters dormancy and the somatic embryo-derived complete plantlet is ready for acclimatization. (**P**) Sterile plants derived from somatic embryos. Ste, seed testa; umbilici, um; em, embryo; en, endosperm; co, cotyledon; ra, radicle; sc, embryogenic callus; rh, rhizome; sc, scales; ab, axillary buds; le, leaf; st, stem; m, month.

Development of *P. polyphylla* plants from zygotic embryos to the one-leaf seedling stage using conventional propagation methods took 18 months, reflecting the rate of development in nature (upper panel, Figure 2). Further, it took 8 months for the radicle and cotyledons to emerge (i.e., for radicle dormancy to be broken) and 10 months for leaf emergence (i.e., for epicotyl dormancy to be broken). In contrast, it took only five months to develop single-leaf plantlets from embryogenic calli (bottom panel, Figure 2). After the apex of the globular embryos became differentiated (Figure 2I), germinated embryos were observed with cotyledons and an elongated hypocotyl in the first month (Figure 2J). By the second month, the elongated cotyledons were yellowish in color (Figure 2K), and the radicles elongated and the cotyledons expanded further in the third month (Figure 2L). The base of the hypocotyl, where the root connects with the stem segment, turned white, swelled, and become oval in shape in the fourth month (Figure 2M). No dormancy was observed before the one-leaf emergence stage (Figure 2N). Thus 13 months were saved in comparison with the zygotic embryo-based propagation method.

### 2.2. High Emergence Rates and Healthy Growth of In Vitro Plantlets in the Field

**Figure 3:**
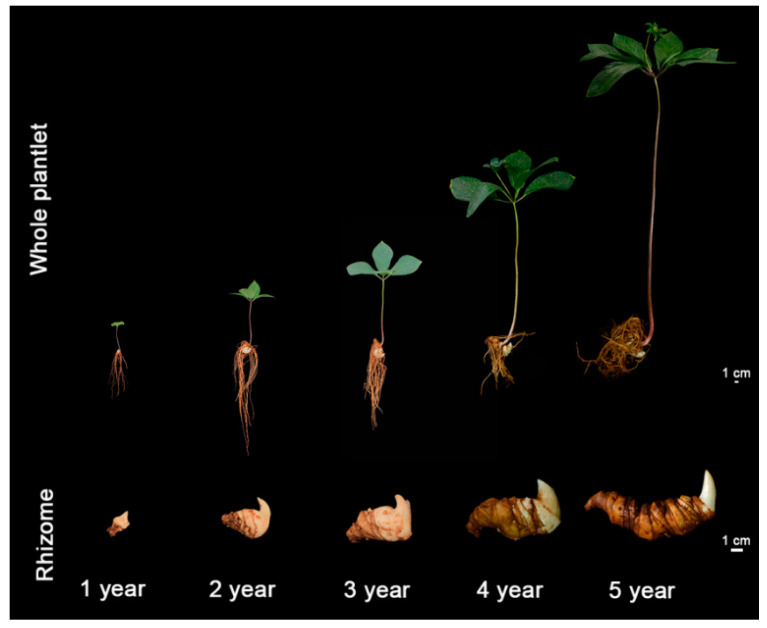
Size comparisons between whole plantlets and rhizomes of *P. polyphylla* during different growth years.

**Figure 4:**
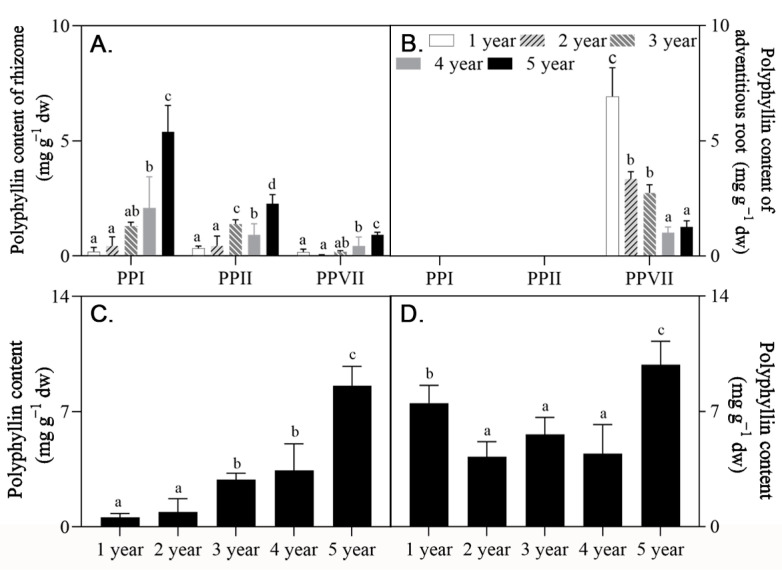
PPI, PPII, and PPVII content in rhizomes (**A**) and adventitious roots (**B**) and total polyphyllin content in rhizomes (**C**) and plantlets (**D**) of *P. polyphylla* with different harvest times (one, two, three, four, and five years old). dw, dry weight. Every value is the mean ± SD (*n* = 5); values with different letters indicate that they had significantly differences between years (*p* ≤ 0.05).

Acclimatization of in vitro plantlets to soil growth conditions is a critical process for any successful SE-based planting industry. To examine the emergence rates of in vitro plantlets during the acclimation period, dormant plantlets were transplanted into soil in the early winter when rhizome dormancy starts (Figure 2O). When *P. polyphylla* naturally sprouted in June, 73.0% of the examined and then non-dormant rhizomes had sprouted. They continued to sprout throughout July and August and reached 94.4% emergence (Table S2). The monitoring of *P. polyphylla* plantlets’ growth in soil was conducted for five years (Figure 3). In the first and second years, plants were mostly observed in the single-leaf stage. In the third to fifth years, the plants were observed with a whorl of three to five leaves and the stem apex had developed into flower primordia. The sepals were green and leaf-like (Figure 4 and Figure S1). These observed morphological changes in in vitro plantlets were the same as those described in wild seedlings [6]. In terms of biomass accumulation, plants showed an annual increase of 3.14 g, 27.80 g, 48.67 g, 95.47 g, and 176.13 g in fresh weight, respectively, over the first five years (Table 1). The average annual growth rates from year two onwards were measured as 785%, 75%, 96%, and 84% (Table 1). All plant parts developed synchronously and the rhizome grew to a mass of 140.44 g, which is good enough for commercial use (Table 1). Taking all results together, in vitro plantlets of *P. polyphylla* exhibited high emergence rates and fast and healthy growth similar to that observed in the field.

### 2.3. High Content of Polyphyllin in In Vitro Plantlets

To evaluate the medicinal properties of in vitro plantlets obtained from *P. polyphylla*, the PPI, PPII, and PPVII contents in rhizomes and adventitious roots were measured for the first five years of growth (Figure 4). All the three polyphyllins were identified in rhizomes but only polyphyllin VII was found in adventitious roots. The three polyphyllins’ concentrations in rhizomes increased as the plants matured, such that the greatest increases (156%) occurred in the fifth year. At this stage of growth, the value reached 8.57 mg/g DW (Table S3), which exceeded the recommended requirement for medicinal use set by the Chinese Pharmacopoeia [2].

### 2.4. Propagation of Four Candidates of P. polyphylla Cultivars

**Figure 5:**
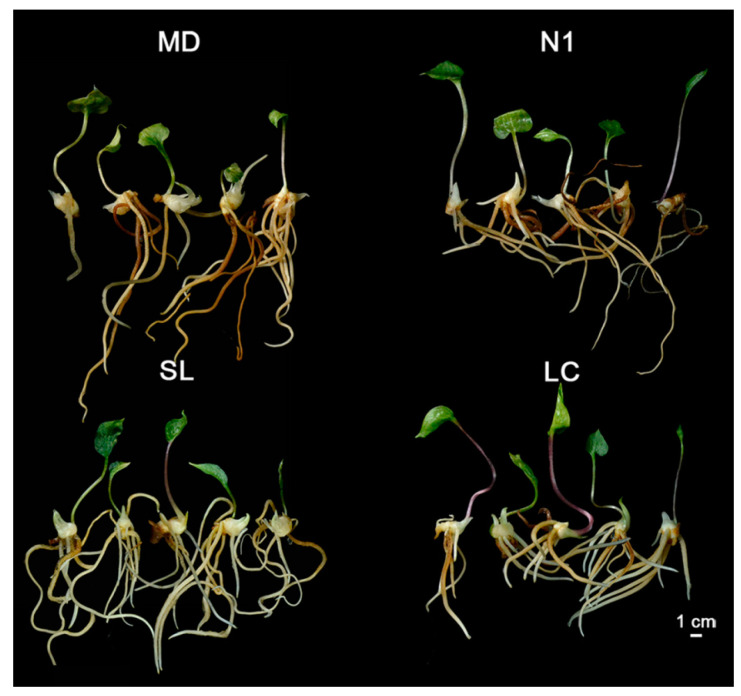
Comparative growth of four *P. polyphylla* accessions MD, N1, SL, and LC after 5 months of incubation under the same in vitro culture conditions.

Rapid accumulation of biomass is one of the major targeted traits for medicinal plant breeding. Using visual inspection, four *P. polyphylla* individuals with strong and fast development (i.e., accessions MD, N1, SL, and LC) were chosen to evaluate the novel production mode’s ability to produce *P. polyphylla* cultivars efficiently and effectively (true-to-type). They were propagated through SE and multiplication showed a similar proliferation rate and period as described above (Figure 2). The morphological developments observed in in vitro plantlets were healthy leaves, rhizomes, and adventitious roots with no other aberrations (Figure 5). However, they showed variability in growth even at an early stage of the in vitro culture. Plantlets of N1 and LC showed significant strong leaf growth (Table 2). Moreover, there was evidence of increased variation in biomass accumulation with fresh weight (such that SL > N1 > MD > LC) after growing for two years (five months in culture medium + 19 months in soil) or after transplanting to soil and growing for 19 months (Table 3). Additionally, plantlets of all prospective cultivars showed 6–16 times greater biomass than YUSD, a farm cultivar grown in soil for the same period of time after seed germination (Table 3). These results suggested that the new production method is not only able to maintain the traits of selected individual *P. polyphylla* but can also give *P. polyphylla* plantlets great growth advantages in the early stage of planting.

### 2.5. Characterization of Long-Term Growth in P. polyphylla Cultivar Candidates

**Figure 6:**
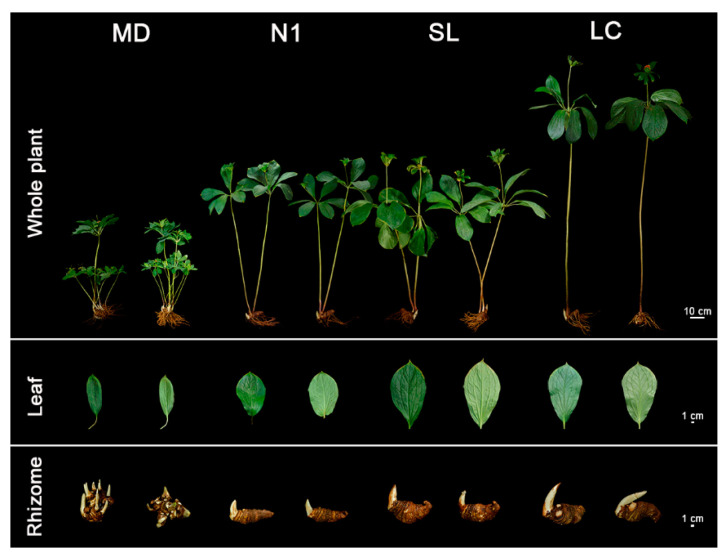
Comparative growth of whole plantlets, leaves, and rhizomes of MD, N1, LC, and SL accessions after five years in the field.

The four *P. polyphylla* cultivar candidates that were propagated using SE were cultivated in the field for five years to assess their growth and development. They showed healthy growth and displayed total morphological features similar to their original accessions (Figure 6). For whole plantlets, MD produced the highest number of aerial shoots, averaging four per rhizome and a maximum of nine. The stems produced were longest in LC, 1.07, 0.80, and 1.04 times longer than those observed in MD, N1, and LC, respectively. This accession is known to produce long stems and the in vitro growth pattern was consistent with the field characteristics (Figure 6, Table 4). Though MD had the most leaves, their fresh weight was lower, which contributed to their reduced surface area. The leaf shapes of the four cultivars were also different. MD leaves were lanceolate, N1 leaves were broadly ovate, and LC and SL leaves were obovate-elliptical (Figure 6, Table 4). MD is known to be an accession capable of producing polyapical shoots, and multi-axillary buds were observed on in vitro MD rhizomes, averaging 5.5 buds per rhizome with a maximum of 17. The other three cultivars were dominated by mono-axillary buds. The fresh weight of the rhizome of SL was 216.44 g, which was 1.59, 1.54, and 1.16 times those of MD, N1, and LC, respectively (Figure 6, Table 4). Although the rhizome yield in SL was found to be superior to the other three accessions, the rhizomes biomasses of all four accessions met the requirements of medicinal harvesting.

### 2.6. Characterization of Active Gradient Accumulation in Long-Term Growth Cultivar Candidates

**Figure 7:**
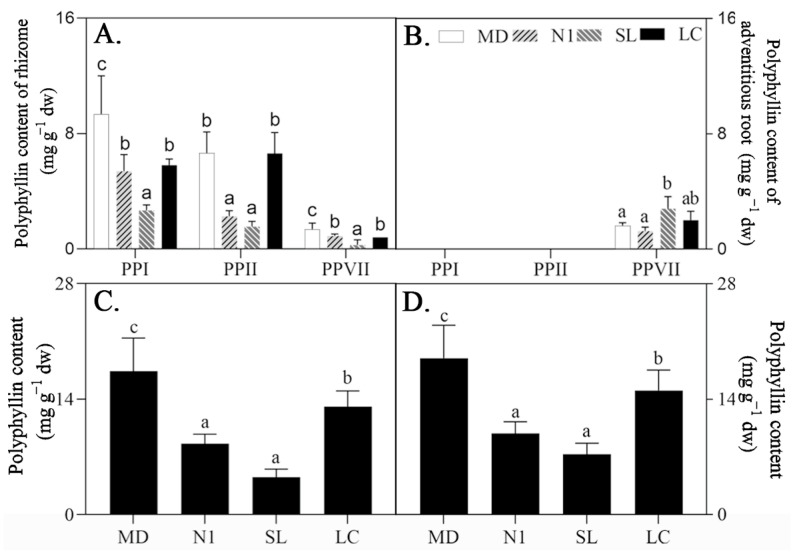
Polyphyllin (PPI, PPII, and PPVII) content in accessions MD, N1, LC, and SL after five years’ growth: polyphyllin content in rhizomes (**A**) and adventitious roots (**B**) and total polyphyllin content in rhizomes (**C**) and in adventitious roots (**D**). dw, dry weight. Every value is the mean ± SD (*n* = 5) and values with different letters indicate significant differences (*p* ≤ 0.05) between accessions.

The polyphyllin content in the four cultivar candidates (accessions) was examined after growth in the field for five years. Four cultivar candidates’ rhizomes contained PPI, PPII, and PPVII; however, PPVII was only discovered in adventitious roots (Figure 7, Table S4). The polyphyllin content in MD, LC, N1, and SL rhizomes was 18.99, 15.07, 9.83, and 7.31 mg/g, respectively. In terms of active ingredients, MD and LC had the highest polyphyllins levels, making them the two top cultivar choices for breeding. Moreover, after a five-year growing period, MD, LC, and N1 accumulated sufficient polyphyllin to exceed the threshold set by the Chinese Pharmacopoeia (0.60%).

## 3. Discussion

The *P. polyphylla* planting industry needs an efficient technique for uniformly producing rhizomes to meet the lucrative demands of pharmaceutical use. In order to produce *P. polyphylla* plantlets, this study established a new production method (innovation platform) based on somatic embryos. Somatic embryos of *P. polyphylla* could be efficiently generated using somatic calli on “filter paper + medium” and the somatic embryos could develop into plantlets within five months. The plantlets’ 94.4% emergence rate and good morphological growth demonstrated excellent adaptation to the soil conditions following in vitro growth. Their development was rapid, and the fifth year in the field showed the highest increase in biomass. Rhizomes accumulated PPI, PPII, and PPVII, and the contents increased as their planting age increased. In the fifth year, we observed the greatest increases in polyphyllin contents to the extent that the requirement for pharmaceutical use was exceeded. This clonal propagation method was successfully applied to four selected *P. polyphylla* individuals, which were candidates for cultivar development. In vitro propagation of the four genotypes resulted in benefits in terms of scale and efficacy and preserved the natural morphological forms and the active ingredient contents of these cultivar candidates. This newly developed innovation platform has great potential to meet the current needs of the *P. polyphylla* traditional Chinese medicine industry.

Proliferation rate is a key factor for a successful planting industry. Even though producing plantlets through somatic embryogenesis is considered to be the most efficient micropropagation technique, different SE culture conditions can result in huge differences in proliferation rates when differentiating from embryogenic calli. Previous studies have shown that filter paper on solid growth medium can improve the aeration of embryogenic calli. Such improved aeration probably reduces ethylene levels, and this could be helpful for embryo maturation and eventually increase the proliferation rate of calli [16]. When cultured on filter paper, somatic embryos of *Gossypium arboreum* L. [17], *Pinus pinea* L. [18], *Anthurium andraeanum* Lind. [19], and *Gossypium stockii* [20] have shown high proliferation rates. In this study, culturing on filter paper increased the *P. polyphylla* SE rate of proliferation by sevenfold, indicating considerable potential for the large-scale production of *P. polyphylla* plantlets.

**Figure 8:**
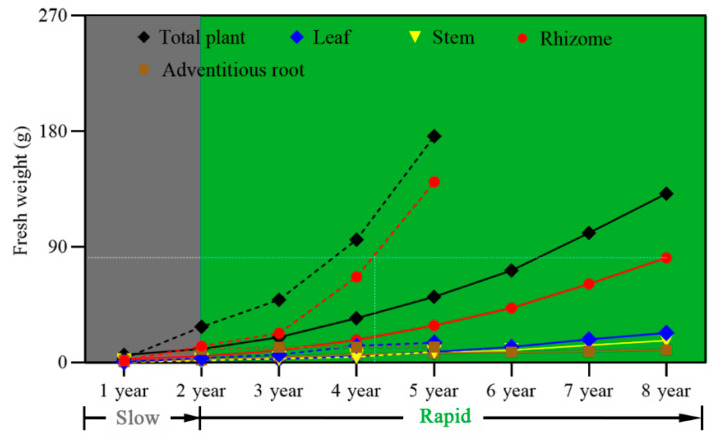
The increase in *P. polyphylla* biomass with time after planting. Solid line, growth curve for seedlings, data from Chen and Liu [22,23]. Dotted line, growth curve for plantlets, data from this study. Grey area, slow growth zone. Green area, rapid growth zone. White dotted line, comparison of rhizome growth between seedlings and plantlets.

With traditional planting, *P. polyphylla* needs to grow in the field for as long as eight years before rhizome harvesting (Figure 8), which is a major problem for production efficiency. The long growing period results from dormancy release needing two winters [6,7] and the general slow growth of the rhizome [21]. Production of *P. polyphylla* plantlets through SE skipped dormancy release and thus saved 13 months in the production timetable, which means a 22.5% increase in time efficiency. One additional benefit of in vitro culture conditions is the provision of abundant nutrients, which resulted in the axillary buds of plantlets being larger than those of seedlings (Figure 2F,N). This response could lead to stronger and faster-growing plantlets after transplantation into soil. Accordingly, the growth curve of the plantlets generated via SE was in the fast growth zone [22,23] (Figure 8). The time possibly saved for rhizome harvesting can be estimated as at least three years (Figure 8). Therefore, this new industry method could represent an increase in time efficiency of 37.5%.

The heterogeneous raw materials resulting from the interspecific hybridization of *P. polyphylla* are a severe problem for the planting industry [24,25]. In contrast, we show that clonal propagation using SE resulted in a high proliferation rate, which is advantageous for both genetic resource conservation—because of the suitability of SE for cryopreservation [26]—and the provision of homogenous raw materials for medicinal use. Application of SE for the clonal propagation of four selected wild *P. polyphylla* individuals consistently produced plants with beneficial features in terms of growth or the content of active ingredients.

## 4. Materials and Methods

### 4.1. Plant Material and Embryogenic Callus Conversion to Plantlets

In vitro plantlets were generated from selected individuals of four elite wild species accessions collected from Mouding (MD), Wuding (N1), Shilin (SL), and Lancang (LC) counties in Yunnan province, China. Among them, the MD species is a typical “dwarf stem” plant and the LC species a typical “high stem” plant. The N1 and SL accessions were the other best accessions in the same year, with heights between those of the LC and MD accessions.

Embryogenic calli were inoculated in half-strength Murashige and Skoog (MS) medium containing 30 g L^−1^ sucrose, 3 g L^−1^ Phytagel™, 0.2 g L^−1^ hydrolyzed casein, and l g L^−1^ polyvinyl pyrrolidone in a 9 cm diameter Petri dish. The pH of the media was adjusted to 5.7–5.8 (using 1 M HCl or 1 M KOH) prior to autoclaving at 121 °C for 15 min. Embryogenic calli were picked in a Petri dish with filter paper (Whatman; cat. no. ROHU-SQDLLZZ122) + medium. The control culture was placed directly in the growth medium only. All of the cultures were maintained at 23 °C with a 16/8 h (day/night) photoperiod, a photo flux density of 300 μmol m^−2^ s^−1^, and 60% relative humidity. The number of germinated somatic embryos per gram of embryogenic callus was recorded after 30 days of culture. After six months of plantlet growth, the fresh weights of leaves, rhizomes, stems, and adventitious roots were recorded and analyzed statistically.

A farm cultivar YUSD was used as a zygotic embryo control. The YUSD seeds were obtained from the Lancang Pengbo Biological Pharmaceutical Corporation, China, in 2016. Seeds of YUSD control plants were sown in the soil in November 2016. Cultivation methods and conditions were identical for control plants and clonally propagated plantlets. Statistical analysis was performed with a sample of seedlings that sprouted two years after seeding. Data on the biomass of the seedlings were collected from November to December 2018.

### 4.2. Field Transfer of Regenerated Plants

The plantlets that entered the dormant stage were removed from the culture medium and washed gently with distilled water to remove all remnants of agar. Then, they were transplanted into breeding bags (12 cm diameter and 18 cm height) with soil (25% peat, 25% humus, and 50% red soil) in November 2016. The planting distance was 7 × 7 cm. The place of cultivation was Greenhouse No. 12, Kunming Institute of Botany, Chinese Academy of Sciences. The shading density, watering and manual weeding, and soil matrix were the same for all greenhouses during the entire duration of planting.

### 4.3. Emergence Rate after Transplanting

Sprouting, nonsprouting, and death rates for *P. polyphylla* were recorded during June 2017 and calculated as follows:

Sprouting rate = number of sprouting plants/total number of transplanted plants;

Nonsprouting rate = number of nonsprouting plants/total number of transplanted plants;

Death rate = number of dead plants/total number of transplanted plants.

The mergence rate for *P. polyphylla* was recorded in August 2017 and calculated as follows:

Emergence rate = number of emergent plants/total number of transplanted plants.

### 4.4. Biomass Measurements

The biomass was harvested before the dormant period (MD, November; N1, November; SL, November; LC, February). After transplantation, N1, MD, SL, and LC plants from one to five years old were selected randomly. Soil was removed with clean water and the roots were kept intact. The fresh weights of rhizomes, leaves, stems, and adventitious roots were measured. The numbers of stems and axillary buds and the stem length were also measured during the fifth year. Ten replicates of each treatment for each cultivar were considered for biomass measurements.

### 4.5. Extraction and Analysis of Saponins

To prepare the crude extract, clean rhizomes and adventitious roots of *P. polyphylla* were shade-dried and finally ground to a fine powder. A sample of the powder (0.2 g) was dissolved in 10 mL of 100% ethanol and extracted using ultrasonication for 30 min. Five biological replicates were used for analysis. After extraction, quantification of saponin was carried out using a standard assay, and high-performance liquid chromatography (HPLC) analysis was performed for the identification, as described previously (Chinese Pharmacopoeia Commission, 2020). The supernatant was transferred to different tubes and filtered through a 0.45 μm filter. The filtrate was employed for the identification of the saponin content using HPLC (Agilent 1260, Agilent Technologies, USA). The conditions used during HPLC analysis were as follows: column—C18, 4.6 mm × 100 mm; detection wavelength—203 nm; column temperature—30 °C. The gradient mobile phase consisting of component C (water) and component D (acetonitrile) was used. The mobile phase gradient elution program was as follows: 0–40 min, 30–60% A, 70–40% B; 0–50 min, 60–30% A, 40–70% B. The flow rate was kept constant at 0.8 mL min^−1^. The targeted bioactive compounds, such as PPI, PPII, and PPVII, were measured. PPI and PPII are diosgenin saponins, while PPVII are pennogenin saponins. To quantify steroidal saponin levels, three steroidal saponins were quantified using external standards. The steroidal saponins were prepared as 0.4 mg mL^−1^ standard solutions in methanol, which were sequentially diluted to five concentrations and subjected to UPLC-MS analysis. The three steroid saponins were identified by their retention time relative to the corresponding standard compounds. The content of each component was measured based on the peak areas of the sample and calibration curves.

### 4.6. Statistical Analysis

Analysis of the data was carried out using analysis of variance (ANOVA) in SPSS Statistics 19.0 software (IBM Corporation, Amenk, NY, USA). The figures were generated using GraphPad Prism 6 (GraphPad Software, San Diego, CA, USA).

## 5. Conclusions

The current study demonstrated that SE is an efficient and stable method for the propagation and cultivation of *P. polyphylla* plantlets. In detail, the main conclusions are:

(1) Somatic embryos of *P. polyphylla* were efficiently produced from somatic calli. The proliferation rate on “filter paper + medium” was sevenfold higher than that on medium only;
(2) Within five months, the somatic embryos became one-leaf plantlets. When comparing plants derived from seeds with plants derived from somatic embryos, the latter needed 13 months less to reach the same development stage;
(3) With a 94.4% emergence rate and good morphological development, the SE-derived plantlets showed an outstanding ability to adapt to soil conditions;
(4) After five years’ growth in the field, the plantlets from somatic embryos accumulated enough biomass for commercial use and their rhizomes accumulated PPI, PPII, and PPVII at contents that exceeded the minimum threshold set by the Chinese Pharmacopoeia. In comparison to conventional seedlings, the growth innovation platform using SE saved three years’ (37.5%) production time;
(5) Four elite wild *P. polyphylla* individuals were selected and clonally propagated, resulting in plants with beneficial growth patterns and the desired active ingredient composition. These elite individuals could serve as candidates for commercial variety development. After the assessment of plant growth and active ingredient composition, the LC accession showed great potential to become an elite cultivar due to its superior traits.

## Figures and Tables

**Table 1:** Fresh weight of whole plantlets, leaves, stems, rhizomes, and adventitious roots (g) of *P. polyphylla* in different growth years.

Year after Transplanting	Whole Plantlets	Leaves	Stems	Rhizomes	Adventitious Roots
1	3.14 ± 0.88 ^a^	0.29 *±* 0.10 ^a^	0.19 *±* 0.07 ^a^	1.27 *±* 0.25 ^a^	1.39 *±* 0.53 ^a^
2	27.80 ± 8.07 ^b^	3.37 *±* 1.08 ^b^	1.59 *±* 0.85 ^b^	12.78 *±* 4.94 ^a,b^	10.06 *±* 2.50 ^b^
3	48.67 ± 9.92 ^c^	6.00 *±* 2.17 ^c^	2.87 *±* 1.16 ^c^	22.74 *±* 8.22 ^b^	11.78 *±* 4.38 ^b^
4	95.47 ± 15.64 ^d^	12.82 *±* 1.60 ^d^	4.31 *±* 1.07 ^d^	66.60 *±* 14.88 ^c^	11.75 *±* 2.47 ^b^
5	176.13 ± 46.91 ^e^	15.50 *±* 3.07 ^e^	8.22 *±* 1.83 ^e^	140.44 *±* 35.60 ^d^	11.97 *±* 6.28 ^b^

Note: Values are means ± SD (*n* = 10). Different letters above the values indicate significant differences (*p* ≤ 0.05) between years of growth.

**Table 2:** Fresh weight of leaves, rhizomes, stems, and adventitious roots (g) of MD, N1, SL, and LC after 5 months of incubation under the same in vitro culture conditions.

Accession	Leaves	Stems	Rhizomes	Adventitious Roots
MD	0.02 ± 0.01 ^a^	0.03 ± 0.02 ^a^	0.19 ± 0.11 ^a^	0.33 ± 0.13 ^a^
N1	0.06 ± 0.03 ^b^	0.04 ± 0.02 ^a^	0.29 ± 0.15 ^a^	0.34 ± 0.19 ^a^
SL	0.02 ± 0.01 ^a^	0.03 ± 0.01 ^a^	0.34 ± 0.11 ^a^	0.31 ± 0.23 ^a^
LC	0.06 ± 0.01 ^b^	0.05 ± 0.02 ^a^	0.23 ± 0.13 ^a^	0.26 ± 0.13 ^a^

Note: Values are means ± SD (*n* = 10). Different letters above the values indicate significant differences (*p* ≤ 0.05) between accessions.

**Table 3:** Fresh weight of whole plantlets, leaves, stems, rhizomes, and adventitious roots (g) of *P. polyphylla* accessions grown in vitro (MD, N1, SL, LC) and YUSD (farm cultivar) grown in soil at the end of year 2.

Accession	Whole Plantlets	Leaves	Stems	Rhizomes	Adventitious Roots
YUSD	4.16 ± 2.51 ^a^	0.41 ± 0.21 ^a^	0.27 ±0.10 ^a^	2.07 ± 1.11 ^a^	1.40 ± 1.16 ^a^
MD	23.06 ± 10.10 ^b^	2.00 ± 1.16 ^b^	1.53 ±0.86 ^b^	12.57 ± 6.36 ^b^	6.96 ± 2.63 ^b^
N1	27.80 ± 8.07 ^b^	3.37 ± 1.08 ^b^	1.59 ± 0.85 ^b^	12.78 ± 4.94 ^b^	10.06 ± 2.50 ^b^
SL	49.61 ± 10.15 ^c^	8.74 ± 2.50 ^c^	4.54 ± 2.10 ^c^	24.52 ± 8.24 ^c^	11.81 ± 3.29 ^d^
LC	66.33 ± 20.81 ^d^	7.42 ± 1.70 ^c^	3.57 ± 1.30 ^c^	37.83 ± 13.08 ^d^	17.52 ± 5.55 ^c^

Note: Values are means ± SD (*n* = 10). Different letters above the values indicate significant differences (*p* ≤ 0.05) between accessions.

**Table 4:** Fresh weight of whole plantlets, leaves, stems, rhizomes, and adventitious roots (top panel); numbers of stems and axillary buds; and stem lengths (bottom panel) for MD, N1, LC, and SL accessions after five years’ growth in the field.

Accession	Whole Plantlets (g)	Leaves (g)		Stems (g)	Rhizomes (g)		Adventitious Roots (g)
MD	163.65 ± 57.58 ^a^	20.75 ± 6.35 ^a,b^		10.44 ±3.60 ^a^	136.27 ± 47.22 ^a^		21.16 ± 13.82 ^a^
N1	176.13 ± 46.91 ^a,b^	15.50 ± 3.07 ^a^		8.22 ± 1.83 ^a^	140.44 ± 35.60 ^a^		11.97 ± 6.28 ^a^
SL	273.59 ± 57.59 ^c^	30.50 ± 6.54 ^c^		10.22 ± 2.21 ^a^	216.44 ± 49.70 ^b^		16.43 ± 7.16 ^a^
LC	236.96 ± 54.87 ^b,c^	22.50 ± 6.45 ^b,c^		15.11 ± 3.75 ^b^	185.93 ± 57.44 ^b^		13.42 ± 5.66 ^a^
	Number of stems		Number of axillary buds			Stem length (cm)	
MD	4.00 ± 2.36 ^b^		5.50 ± 5.5 ^b^			68.97 ± 18.53 ^a^	
N1	2.00 ± 0.47 ^a^		1.00 ± 0 ^a^			79.77 ± 12.06 ^a^	
SL	2.70 ± 1.05 ^a,b^		2.40 ± 2.01 ^a^			70.47 ± 13.85 ^a^	
LC	1.10 ± 0.32 ^a^		1.10 ± 0.32 ^a^			143.77 ± 12.96 ^b^	

Note: Values are means ± SD (*n* = 10). Different letters above the values indicate significant differences (*p* ≤ 0.05) between accessions.

## Data Availability

All the data supporting the findings of this study are included in this article.

## Supplementary Materials

The following supporting information can be downloaded at: https://www.mdpi.com/article/10.3390/plants12162983/s1, Figure S1: Structure of *P. polyphylla* after 5 years; Table S1: The effect of filter paper on SE induction frequency; Table S2: Plantlet sprouting, nonsprouting, and death rates (%). n = 300; Table S3: Polyphyllin contents in rhizomes and adventitious roots (mg g−1 dw) of *P. polyphylla* at different harvest times (1, 2, 3, 4, and 5 years old); Table S4: PPI, PPII, and PPVII content in rhizomes and adventitious roots (mg g−1 dw) of MD, N1, LC, and SL plants after 5 years.
