# Supplementary material for: Clonal Propagation and Assessment of Biomass Production and Saponin Content of Elite Accessions of Wild Paris polyphylla var. yunnanensis
Source: Plants (Basel). 2023 Aug 18;12(16):2983. doi: 10.3390/plants12162983 (PMC10459934; doi:10.3390/plants12162983)
Supplement: Supplementary file 1 [file plants-12-02983-s001.zip › Supplementary Material.pdf]

plants

Supplementary Material

Clonal propagation and assessment of biomass production and saponin content of elite accessions of wild *Paris polyphylla* var. *yunnanensis*

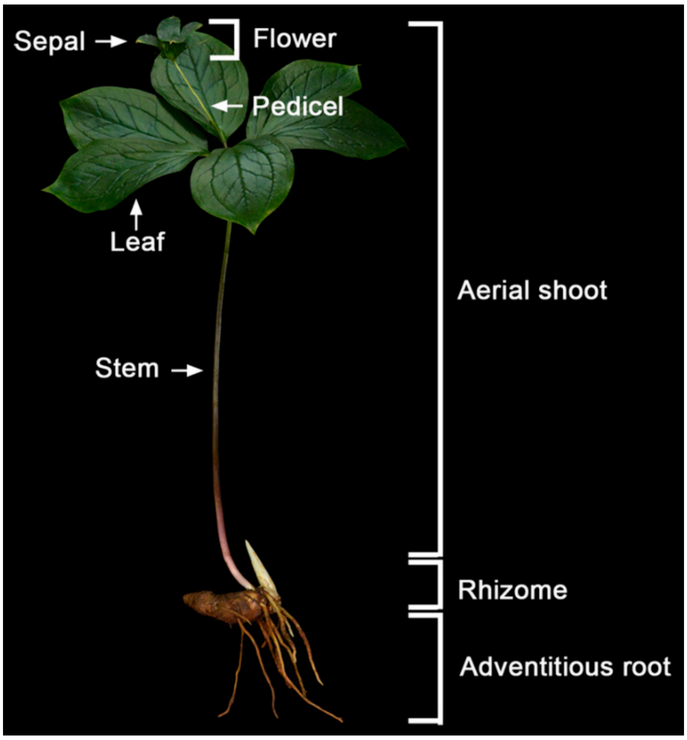

Figure S1. Structure of *P. polyphylla* of 5 years.

Table S1. The effect of filter paper on SE induction frequency.

	Medium	Filter paper + medium
Number of somatic embryos	9.00 ± 3.56	63.10 ± 31.10*

Note: Values are means ± SD (n = 10). Asterisk above the values indicate significant differences ($P \leq 0.05$) between different treatments.

Table S2. Rate of plantlets sprouting, nonsprouting and death (%). *n* = 300.

Sprouting rate	Nonsprouting rate	Death rate
73.9%	20.5%	5.6%

Table S3. Polyphyllins contents in rhizomes and adventitious roots (mg g^{-1} dw) of *P. polyphylla* in different harvest times (1, 2, 3, 4 and 5 years old).

Year after transplanting	Rhizomes			Total content of rhizome	Adventitious roots	Total content
	PPI	PPII	PPVII		PPVII	
**1**	0.18 ± 0.18[a]	0.33 ± 0.11[a]	0.17 ± 0.13[a]	0.68 ± 0.23[a]	6.93 ± 1.24[c]	7.61 ± 1.07[b]
**2**	0.44 ± 0.39[a]	0.44 ± 0.43[a]	0.03 ± 0.02[a]	0.90 ± 0.81[a]	3.35 ± 0.31[b]	4.25 ± 0.92[a]
**3**	1.29 ± 0.17[ab]	1.39 ± 0.19[c]	0.19 ± 0.04[ab]	2.87 ± 0.39[b]	2.73 ± 0.75[b]	5.60 ± 1.03[a]
**4**	2.08 ± 1.36[b]	0.91 ± 0.48[b]	0.43 ± 0.39[c]	3.42 ± 1.62[b]	1.01 ± 0.25[a]	4.43 ± 1.77[a]
**5**	5.39 ± 1.15[c]	2.27 ± 0.39[d]	0.92 ± 0.12[c]	8.57 ± 1.17[c]	1.26 ± 0.27[a]	9.83 ± 1.42[c]

Note: Values are means ± SD (n = 5). Different letters above the values indicate significant differences ($P \leq 0.05$) between different treatments.

Table S4. PPI, PPII and PPVII content in rhizomes and adventitious roots (mg g^{-1} dw) of MD, N1, LC and SL in 5 years.

Accession	Rhizomes			Total content of rhizome	Adventitious roots	Total content
	PPI	PPII	PPVII		PPVII	
MD	9.23 ± 2.64^{c}	6.64 ± 1.46^{b}	1.37 ± 0.42^{c}	17.36 ± 4.02^{c}	1.63 ± 0.21^{a}	18.99 ± 3.99^{c}
N1	5.39 ± 1.15^{b}	2.27 ± 0.39^{a}	0.92 ± 0.12^{b}	8.57 ± 1.17^{a}	1.26 ± 0.27^{a}	9.83 ± 1.42^{a}
SL	2.68 ± 0.38^{a}	1.57 ± 0.35^{a}	0.27 ± 0.37^{a}	4.50 ± 1.02^{a}	2.80 ± 0.85^{b}	7.31 ± 1.37^{a}
LC	5.80 ± 0.43^{b}	6.61 ± 1.46^{b}	0.81 ± 0.00^{b}	13.07 ± 1.91^{b}	2.00 ± 0.63^{ab}	15.07 ± 2.47^{b}

Note: Values are means ± SD ($n = 5$). Different letters above the values indicate significant differences ($P \leq 0.05$) between different treatments.
